# Factors associated with quality of life for cystic fibrosis family caregivers

**DOI:** 10.1007/s44192-023-00046-1

**Published:** 2023-10-16

**Authors:** Whitney Nesser, Scott Snyder, Kimberly A. Driscoll, Avani C. Modi

**Affiliations:** 1 Department of Applied Clinical and Educational Sciences, Indiana State University, 401 N. 7th Street, Room 302B, Terre Haute, IN 47809 USA; 2 School of Education, University of Alabama at Birmingham, Birmingham, AL USA; 3 Department of Clinical and Health Psychology, University of Florida, Gainesville, FL USA; 4 Center for Adherence and Self-Management, Cincinnati Children’s Hospital Medical Center, Cincinnati, OH USA

## Abstract

Cystic Fibrosis (CF) is a genetic and chronic disease affecting 32,100 people in the United States as of 2021, with a life expectancy of 56 years for people with CF (PwCF) born between 2018 and 2022. While there is extensive literature about cystic fibrosis, there are few studies examining the complexity and challenges experienced by family caregivers for PwCF. The aim of this study was to examine the Caregiver Quality of Life Cystic Fibrosis (CQOLCF) scale using data (N = 217) from two separate studies that used the scale to determine if its items represent multiple factors relevant to CF family caregiver QoL. Factor analysis was conducted on the Seven distinct factors were found with analysis of the CQOLCF. Factors were Existential Dread (12%), Burden (11%), Strain (7%), Support (7%), Positivity (6%), Finance (5%) and Guilt (3%). Study findings indicated it is important for healthcare providers and researchers who use the CQOLCF to be knowledgeable and aware of the multiple factors associated with quality of life in this population in addition to an overall quality of life score.

## Introduction

Cystic Fibrosis (CF) is a genetic and chronic disease affecting 32,100 people in the United States as of 2021, with an increased life expectancy of at least 56 years for people with CF (PwCF) born between 2018 and 2022, primarily because of improvements in genetic testing and therapeutic advances [1–3]. Of particular note, two pivotal trials directly impacted and improved life expectancy through the initiation of a highly effective modulator known as ETI (Elexacaftor/Tezacaftor/Ivacaftor) in 92% to 93% of the CF population [4–6]. These modulators have transformed CF care so that for PwCF, lung function has improved substantially, with fewer hospitalizations, and lower mortality [7]. While remarkable advances continue with treatment and prognosis allowing the life expectancy of PwCF to be comparable to the general population [7], CF remains a genetic, chronic disease with no cure. Newborn screening helps to ensure a CF diagnosis within the within the first 6 months of life [8], and concurrently, a CF diagnosis is also when family caregivers become responsible for treatment complexity management, resulting in caregiver burden and other associated health outcomes dependent upon consistent access to care [9, 10]. While there is extensive literature about CF, there are few studies examining the complexity and challenges experienced by family caregivers for PwCF [11–15].

The complexity of CF places tremendous responsibility on and unique challenges for CF caregivers. In 2016, the International Committee on Mental Health in Cystic Fibrosis developed clinical practice guidelines for PwCF and their caregivers [16]. These guidelines for caregivers encompass prevention, screening, and assessment for depression and anxiety with data supporting these recommendations for improved quality of life (QoL) and health. All CF Centers in the United States have now implemented annual mental health screenings for PwCF beginning at age 12 and for parent caregivers of PwCF, infants through adults [17]. Among the CF caregiving population, high rates of depression and anxiety [17, 18] as well as burden [19] have been identified, yet it is unclear how CF caregivers experience other QoL dimensions.

Caregiver QoL has several dimensions including psychological, physical, social, and environmental [20–23] and has been established as clinically important to improve patient outcomes, early detection of caregiver health risks, targeted interventions, and provision of resources for caregivers [11, 14, 24–29]. Caregiver QoL literature is extensive, particularly in cancer and dementia populations [30–36], but there is limited literature specific to CF caregiver QoL [11, 13] and no study or measure to date has comprehensively assessed the multidimensional nature of caregiver QoL. The Caregiver Quality of Life for Cystic Fibrosis (CQOLCF) scale represents the only QoL scale specific to CF caregivers [11, 13, 22, 37, 38].

The CQOLCF was developed based on the Caregiver Quality of Life Index—Cancer (CQOLC), a scale measuring the quality of life for caregivers of people with cancer [39]. Items were reviewed by a CF expert panel and revised for CF caregivers resulting in 35 items and Likert scale response options (0 = Not at all to 4 = Very much), and a total score ranging from 0 to 140. Details of the initial development and validation of the CQOLCF are available in a previous publication [22]. In the Boling, Macrina, & Clancy study (2003), convergent validity of the CQOLCF with the Beck Depression Inventory and Medical Outcomes Short Form 12 (SF-12) indicated the CQOLCF was positively correlated with depression for CF caregivers [22]. In the Driscoll, Montag-Leifling, Acton, & Modi study (2009), caregiver depressive symptoms predicted caregiver QoL based on the CQOLCF [14]. Although the CQOLCF was initially developed to represent multiple factors that impact CF family caregiver quality of life, it only yields a total score with no guidance for what the range of scores indicate [22, 39]. The aim of this study was to examine the CQOLCF to determine if its items represent multiple factors relevant to CF family caregiver QoL.

## Methods

### Participants and procedures

This study was a secondary analysis performed with data collected from two prior studies that used the CQOLCF [14, 22]. In the Boling et al. study [22], the CQOLCF was developed and validated to assess family caregiver QoL in the CF population. The overall goal of the Boling et al. study was to modify the CQOLC for CF family caregivers, as well as to assess its split-half reliability, internal consistency, validity, and relationship of QoL to patient disease severity. In the Boling et al. study [22], caregivers were predominantly Caucasian (97%), female (81%), married (69%), and employed (61%) with a mean age of 38 years. The age range for those with CF was 1 to 31 years (*M* = 10.8 ± 7.2). A trend of decreasing caregiver QoL was seen between the disease severity categories. Mean caregiver scores as measured by the CQOLCF ranged from 87.90 to 101.82. CQOLCF means did not change significantly over disease severity categories. Findings indicated the CQOLCF to be a valid and reliable instrument in a sample of CF caregivers, and suggest that caregiver QoL inversely correlates with a marker of disease severity.

In a later study [14], the CQOLCF was used to assess the relationship between depression, anxiety, and QoL among parent caregivers of children with CF. In this study [14], the majority of caregivers were Caucasian (98.2%) and female (82%), with ages ranging from 21 to 50 years (female mean age 37.60; male mean age 40.58). The age range for those with CF, was 1 to 17 years. Mean scores on the CQOLCF were 97.74 for female caregivers and 100.65 for male caregivers, with better child lung functioning associated with fewer depressive symptoms and better QoL in female caregivers. These findings suggest that lung functioning may play a greater role in determining caregiver mood as most of the morbidity and mortality in CF is due to respiratory infection and disease. As measured by the CQOLCF, caregiver QoL was associated with both depressive and anxious symptoms, likely due to QoL being a broad construct that captures psychological functioning.

### Statistical methodology

Data derived from two studies with a total of 217 subjects were merged and submitted to orthogonal principal axis factoring to identify independent dimensions of caregiver QoL evident within the measure. The sample sizes for each study were insufficient to allow for independent factor analyses while 200 subjects meet the minimum criteria proposed by several authors [40–42]. Several prior studies examining the underlying structure of the CQOLC have used principal components analyses with orthogonal rotations [43–45]. Such analyses are effective in identifying orthogonal (uncorrelated) linear composites of variables that are effective in maximizing the total explained variance within a data set. However, the intent of this study was to explore the underlying factor structure of the CQOLCF that is explained through shared variance amongst items. To get an appropriate perspective on the underlying structure of the CQOLCF an exploratory factor analysis, rather than a confirmatory factor analysis, was deemed most appropriate for exploring the dimensionality of the CQOLCF. Principal components analysis is appropriate for identifying linear components that most efficiently represent the total variance in a scale. Factor analysis is more appropriate for identifying underlying dimension by focusing only on the shared variance amongst items. Therefore, for the purposes of this study to identify orthogonal underlying conceptual dimensions, principal axis factor analysis was used to analyze the CQOLCF. Kaiser criterion was used to determine the number of factors to retain in principal components analyses and factor analysis [46]. This study sought to examine the range of factor solutions resulting from the application of the Kaiser and parallel analysis approaches to factor analysis. While different factor identification approaches were used, all approaches used a communality criterion of greater than 0.32 (greater than 10% of the variance in the item is accounted for by the factors) for selecting factor analysis items. The analyses of this study sought to clarify the underlying dimensions evident within the CQOLCF scale.

## Results

Data for the CQOLCF were available for a total of 217 adult caregivers of PwCF [14, 22]. The combined samples were almost exclusively Caucasian (97% and 98%). The coefficient alpha internal consistency for all 35 items across participants was 0.92.

### Analysis one

The factor analysis that applied the Kaiser criteria yielded a seven-factor solution accounted for 54.9% of the shared variance after four items presented with communalities of less than 0.33 (items 3, 12, 22 and 27) were excluded from the factor analysis. Factors identified were Existential Dread (12.7%), Burden (9.5%), Strain (10.1%), Support (8.0%), Positivity (4.9%), and Finances (5.4%). The finance factor was comprised of only two items. A seventh factor (Guilt), explaining 4.3% of the variance, was retained for discussion purposes since it was only represented by a single item. The item was orthogonal to other factors (3%), met the inclusion criteria, and represented a dimension that, due to the genetic basis of CF and responses from parents in the two studies merged for this analysis, may be important for subsequent examinations of caregiver QoL in those providing care for descendants with an inherited disease. Items had to have a factor loading of above 0.475 to be retained. This assured the importance of the construct to the item and resulted in no item being removed because of its cross-loading on two factors. One item, “I feel under increased mental strain” loaded on existential dread and strain. Due to the larger loading on “Strain” the item was retained there despite its complex loading.

Table 1 includes the factor structure for the scale. The Kaiser–Meyer–Olkin (KMO) was 0.93 and Bartlett’s test was significant indicating factorability of the data. Coefficient alpha levels indicated acceptable levels of internal consistency for six of the factors (alpha > 0.7) and marginal internal consistency (0.63) for the sixth factor.

**Table 1:** Factor Analysis of CQOLCF items with a Two Factor Solution

	Factor	
	1	2
I feel under increased mental strain	0.789	
It bothers me, limiting my focus day-to-day	0.779	
I am discouraged about the future	0.755	
My level of stress and worry has increased	0.737	
The need to manage my loved one's symptoms/illness is overwhelming	0.734	
I feel sad	0.730	
I feel nervous	0.710	
The responsibility I have for my loved one's care at home is overwhelming	0.709	
My daily life is imposed upon	0.684	
I feel frustrated	0.658	
It bothers me that my daily routine is changed	0.621	
My sleep is less restful	0.611	
It bothers me that my priorities have changed	0.609	
I worry about the impact my loved one's illness has had on my other children or other family members	0.604	
I fear the adverse effects of treatment on my loved one	0.602	
My economic future is uncertain	0.472	
I am satisfied with the support I get from my family		0.679
Family communication has increased		0.674
I get support from my friends and neighbors		0.674
Coefficient alpha	0.93	0.72

### Analysis two

Examination of the parallel analysis resulted in the identification of a two-factor solution best suited to the data (Table 2). Eigenvalues revealed a two-factor solution using principal component analysis or principal axis factoring. For the principal component analysis, only the first two factors of the CQOLCF factor analysis were greater than the eigenvalues generated from the factor analysis of a random set of responses from a sample of similar size. For the principal axis factoring parallel analysis, only the first two eigenvalues estimated from the parallel analysis were above 1.0. This indicated that subsequent factors explained less variance than would be expected for a single item.

**Table 2:** Factor Analysis of CQOLCF items with a Seven Factor Solution

	Factor						
	1	2	3	4	5	6	7
I fear my loved one will die	0.690						
I feel sad	0.641						
I am discouraged about the future	0.628						
It upsets me to see my loved one deteriorate	0.618						
I feel nervous	0.585						
I fear the adverse effects of treatment on my loved one	0.464						
It bothers me that I need to be available to go to so many of my loved one's appointments		0.663					
It bothers me that my daily routine is changed		0.647					
It bothers me that my priorities have changed		0.585					
My daily life is imposed upon		0.577					
The need to manage my loved one's symptoms/illness is overwhelming		0.539					
It bothers me, limiting my focus day-to-day		0.474					
I worry about the impact my loved one's illness has had on my other children or other family members		0.472					
My sleep is less restful			0.607				
I feel under increased mental strain	0.462		0.529				
My level of stress and worry has increased			0.514				
I am satisfied with the support I get from my family				0.707			
It bothers me that other family members have not shown interest in taking care of my loved one				0.676			
I am satisfied with my sex life				0.505			
I get support from my friends and neighbors							
Family communication has increased					0.608		
I have more of a positive outlook on life since my loved one's diagnosis					0.608		
I have developed a closer relationship with my loved one					0.595		
I am under financial strain						0.743	
My economic future is uncertain						0.618	
I feel guilty							0.544
Coefficient alpha	0.85	0.88	0.82	0.70	0.67	0.71	

When the data were run as a two-factor solution, 15 of the 35 items on the scale yielded communalities of less than 0.33. This included the same four items that misfit in the first analysis reported above, and 11 additional items. Of the remaining 20 items, 17 loaded on the first factor (negative impacts of caregiving) and three loaded on the second factor (positive impacts of caregiving). The first factor had a coefficient alpha of 0.93 and the smaller second factor had a coefficient alpha of 0.72.

Given the internal consistency of the scales, summated scores were computed for each scale. Analyses were conducted using summated scores for the six and two factor solutions to determine whether significant differences in subscale scores existed between the two studies for any of the subscales. Results indicate that significant differences between studies existed for the Existential Dread (p = 0.049, Eta-squared = 0.019) and Positive (p = 0.025, Eta-squared = 0.025) subscales of the six-factor solution. The minor differences for two of eight subscales in average subscale performance between two similar samples of caregivers provided some support for the congruence of subscale performance for caregivers of pediatric patients.

## Discussion

The CQOLCF was originally developed as a unidimensional scale [22] consistent with the initial development of the CQOLC scale that it was, in part, based upon [47]. Theoretical advances have promoted caregiver QoL as a multidimensional scale having comprised of domains such as psychological, physical, social, and environmental [29]. Parallel psychometric and empirical examinations of the CQOLC have provided some support for existence of these four domains within the scale. Principal components analysis has been the prevailing method for examining the underlying structure of the CQOLC in different regions and countries, resulting in an interpretation both in terms of its total score and different combinations of subscales [43, 44, 48–50]. Principal components analysis sought to find the linear combinations of variables that accounted for the greatest total shared variance. These combinations were appropriate for summarizing multiple items in an efficient way, but may not be effective in identifying underlying structures that accounted for shared variance between items. Factor analysis, such as principal axis factoring, was more appropriate for such a purpose. The use of principal components analyses rather than factor analysis, combined with differences in criteria for selecting the most appropriate number of components to retain, and challenges in the translation of the scale to different languages and cultures, resulted in considerable uncertainty existing regarding the validity and stability of the underlying structure of the CQOLCF scale.

The CQOLCF has been translated for use in several countries [51]. To our knowledge, no prior study has been conducted regarding the factor structure of the CQOLCF. As the measure was not constructed to reflect predetermined (theoretical) domains of caregiver QoL, two exploratory orthogonal factor analyses with principal axis factoring were conducted to identify unidimensional constructs represented by items within the scale. Different criterion for determining the proper number of factors resulted in two different potential factor structures for the scale.

The first result based on using the frequently applied eigenvalue criteria resulted in a six-factor solution with a single variable indicating an orthogonal seventh factor. This factor structure can be viewed as reflecting the psychological, social, physical, and environmental components of the prevailing QoL models [21]. Specifically, the psychological dimension was reflected by Existential Dread, Positivity and Guilt factors, the social dimension was reflected by the Support factor, and the environmental dimension was reflected by the Finances factor. Additionally, both the psychological and physical dimensions were reflected by the Strain factor, while Disruption appeared to reflect psychological, environmental and social dimensions. The lack of clear mapping of items onto these four primary dimensions is not surprising because the initial items for the CQOLC, and therefore of the CQOLCF, were not generated or worded in a manner to reflect the four domains that prevail in the literature today.

The second analysis was based on parallel analysis criteria and resulted in a two-factor solution. This factor structure differentiated items by whether they identified the challenges associated with caring for an individual with CF (e.g. Burden) or the benefits of caring for someone with CF (e.g. Positivity). The two-factor solution involved the removal of 15 of the 35 items from the principal axis factoring because of low extraction communalities. This suggested that the two factors did not explain a substantial amount of the variance in about 43% of the items. The orthogonal nature of these factors indicated a respondent’s average score on the Burden subscale was independent of the respondent’s average score on the Positivity subscale. While this solution was the most parsimonious solution, it may be masking a more complex underlying structure that was revealed by the first analysis.

## Limitations

There are noteworthy limitations within this study. The major limitation is not having confirmation that the CQOLCF is still a valid measure given the transformative changes in CF therapeutics in the last 5 years. Yet, with the core domains of QoL included in the CQOLCF, it appears this measure is still relevant for a general overview of CF caregiver QoL and does generate useful information on caregivers. Another limitation is that the total sample size of the combined data set yielded only 200 usable cases. The researchers acknowledge that a greater sample size would have produced more stable factor solutions. While there is some debate about the minimum necessary sample size [52] there is agreement smaller sample sizes result in less reliable factor solutions. An additional limitation of this study is it was based on data gathered only for caregivers of pediatric CF patients. Dramatic changes in therapies have resulted in a marked extension of the lifespan for patients with CF. Many caregivers today are spouses rather than parents. We are uncertain about the factor structure that would emerge from a sample of caregivers for adults with CF.

## Conclusion

The primary implication of this study is that it is important to interpret CQOLCF scale results as multidimensional rather than unidimensional. While the summated score across all 35 items (after reverse-coding for the items that are worded in an opposite direction from most) was internally consistent, there was little evidence to support that all 35 items represent a unidimensional construct. Researchers using the CQOLCF scale are encouraged, if sample sizes are sufficient, to run exploratory factor analyses or confirmatory factor analyses that seek to confirm the structures found in this analysis.

Adequate QoL measurement of caregivers for patients with CF is clinically and practically important. The multidimensional nature of QoL encompasses the psychological, physical, social, and environmental factors which all contribute to the well-being and health outcomes of both CF caregiver and patient. Work is needed not only on the analyses of the factor structure of the existing CQOLCF scale, but also on the development of an improved instrument to ensure relevance and representation of caregiver QoL in the CF population.

## Data Availability

The data that support the findings of this study are not openly available due to reasons of sensitivity and are available from the corresponding author upon reasonable request. Data are located in controlled access data storage accessible by the corresponding author.
